# Single‐Crystalline β‐Ga_2_O_3_ Homoepitaxy on a Near Van der Waals Surface of (100) Substrate

**DOI:** 10.1002/advs.202417436

**Published:** 2025-03-08

**Authors:** Tong Jiang, Hao Wang, Huaze Zhu, Junwei Cao, Xiaoqing Huo, Zhiqing Yang, Junshuai Li, Yaqing Ma, Shengnan Zhang, Xiang Xu, Wei Kong

**Affiliations:** ^1^ Zhejiang University Hangzhou 310027 China; ^2^ School of Engineering Westlake University Hangzhou 310030 China; ^3^ Key Laboratory of 3D Micro/Nano Fabrication and Characterization of Zhejiang Province School of Engineering Westlake University Hangzhou 310030 China; ^4^ Research Center for Industries of the Future Westlake University Hangzhou 310030 China; ^5^ Westlake Institute for Optoelectronics Hangzhou 311421 China; ^6^ Ji Hua Laboratory Foshan 528200 China; ^7^ China Electronics Technology Group Corp 46th Research Institute Tianjin 300220 China

**Keywords:** β‐Ga₂O₃, single‐crystalline growth, twins, Van der Waals epitaxy

## Abstract

Gallium oxide (Ga₂O₃) is a promising wide‐bandgap semiconductor for power devices, offering high breakdown voltage and low on‐resistance. Among its polymorphs, β‐Ga₂O₃ stands out due to the availability of high‐quality, large‐area single‐crystalline substrates, particularly on the (100) surface, grown via melt‐based bulk crystal growth. However, the low surface energy of β‐Ga₂O₃ (100), akin to 2D materials, presents challenges in homoepitaxy, including poor nucleation and twin formation, which hinder its practical application. This study demonstrates the successful homoepitaxial growth of single‐crystalline β‐Ga₂O₃ on (100) substrates using a van der Waals epitaxial approach. By introducing an excess surfactant metal in metal‐rich conditions at high temperature, a growth regime approximate thermal equilibrium is achieved, enhancing adatom diffusion and suppressing metastable twin phases. This adjustment enables the formation of well‐ordered, single‐crystalline nuclei and lateral stitching in a half‐layer‐by‐half‐layer growth mode, similar to 2D material growth. The result is twin‐free, atomically flat, single‐crystal thin films on on‐axis β‐Ga₂O₃ (100) substrates. These findings significantly improve the crystalline quality of epitaxial β‐Ga₂O₃ on (100) substrates, demonstrating their potential for scalable production of high‐performance, cost‐effective β‐Ga₂O₃‐based power devices, and advancing their feasibility for industrial applications.

## Graphical abstract

This study demonstrates the successful homoepitaxial growth of single‐crystalline β‐Ga₂O₃ on on‐axis (100) substrates using a van der Waals epitaxial approach. By employing an excess metallic surfactant at high temperatures, twin‐free, atomically flat, high‐quality thin films are achieved in a half‐layer‐by‐half‐layer growth mode. This approach enhances the crystalline quality, making it a promising method for scalable, cost‐effective β‐Ga₂O₃‐based power devices on (100) substrates.

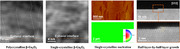

## 1 Introduction

Gallium oxide (Ga₂O₃) has attracted considerable attention due to its promising potential as a wide‐bandgap semiconductor, particularly for power devices that require high breakdown voltage and low on‐resistance.^[^ ^1^, ^2^, ^3^ ^]^ These characteristics position Ga₂O₃ as a strong contender to silicon carbide (SiC), owing to its favorable material properties.^[^ ^4^, ^5^ ^]^ Ga₂O₃ exists in five polymorphic forms, with β‐Ga₂O₃ being the most thermally stable and suitable for melt growth, enabling the production of large‐diameter, low‐cost substrates analogous to silicon.^[^ ^6^, ^7^ ^]^ Currently, 6‐inch β‐Ga₂O₃ wafers in the (100) orientation have been successfully produced, and further advances are expected to increase wafer sizes.^[^ ^8^ ^]^ However, the homoepitaxial growth of β‐Ga₂O₃ on on‐axis (100) substrates is challenging due to the high density of twin boundaries (TBs) and stacking faults (SFs) in the epitaxial layer, which negatively impact carrier mobility and impede device performance.^[^ ^9^, ^10^, ^11^, ^12^, ^13^ ^]^

The (100) surface of β‐Ga₂O₃ exhibits weak interlayer interactions, with a surface energy of 0.34 J m^−^^2^ for the (100) B plane, comparable to the surfaces of 2D materials such as graphite and molybdenum disulfide (MoS₂).^[^ ^14^, ^15^, ^16^ ^]^ Due to these weak interactions, the β‐Ga₂O₃ (100) surface can be exfoliated using methods similar to those used for van der Waals materials.^[^ ^17^ ^]^ The weak interlayer bonding in the (100) plane of β‐Ga₂O₃ presents challenges similar to those encountered in van der Waals epitaxy.^[^ ^18^ ^]^ For example, MoS₂, a typical van der Waals material, can be grown epitaxially on sapphire, but it often forms TBs due to the minimal energy difference between orientations, which is influenced by the higher symmetry of the sapphire substrate.^[^ ^19^ ^]^ Similarly, β‐Ga₂O₃ grown on the (100) plane tends to form 180° in‐plane rotation twins, as the TBs formation energy is extremely low—0.02 J m^−^^2^ for the (100) B orientations—resulting in an infestation of twin defects.^[^ ^10^, ^20^ ^]^ As a result, research on β‐Ga₂O₃ epitaxy and device fabrication on (100) substrates has been limited, and single‐crystalline homoepitaxy without offcut has not been achieved to date.^[^ ^21^ ^]^

In this study, we report the successful twin‐free homoepitaxial growth of β‐Ga₂O₃ on a (100) substrate in a near van der Waals epitaxial manner. By introducing excess indium (In) as a surfactant and employing metal‐rich growth conditions at high‐temperature, we facilitated the decomposition of unstable twin structures and significantly enhanced the surface diffusion length of adatoms. These unconventional conditions promoted the formation of single‐oriented nucleation and lateral stitching in a half‐layer‐by‐half‐layer growth mode, resulting in single‐crystalline epitaxial β‐Ga₂O₃ thin films with atomically flat surfaces. This approach offers a promising pathway for developing high‐performance materials and devices based on (100) β‐Ga₂O₃ substrates, leveraging the advantages of large wafer diameters and the cost‐effectiveness of this material system.

## 2 Results

### 2.1 Surface Properties and Epitaxial Defect Structure in (100) β‐Ga_2_O_3_

**Figure 1:**
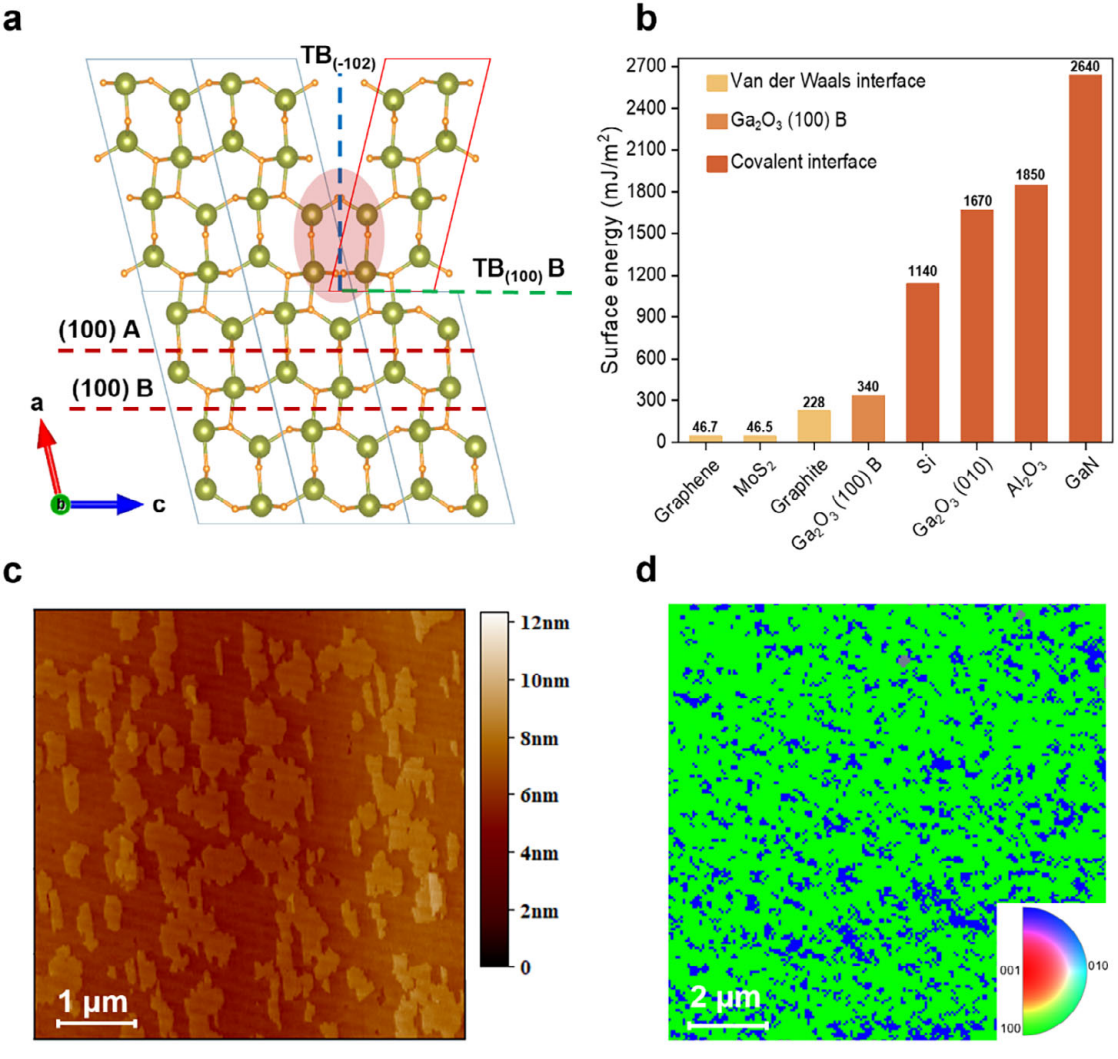
Surface energies and epitaxial defect structures of (100) β‐Ga_2_O_3_. a) Atomic arrangement of β‐Ga_2_O_3_ UC along the [010] axis, showing different surface terminations: “(100) A” and “(100) B”. The dotted green and blue lines represent TB_(100)_ B and TB_(‐102)_, respectively. b) Surface energy comparison for 2D and 3D materials, including graphene,^[^ ^27^ ^]^ MoS_2_,^[^ ^16^ ^]^ graphite,^[^ ^15^ ^]^ silicon,^[^ ^23^ ^]^ β‐Ga_2_O_3_ (100) B,^[^ ^14^ ^]^ β‐Ga_2_O_3_ (010),^[^ ^14^ ^]^ Al_2_O_3_,^[^ ^24^ ^]^ and GaN.^[^ ^25^, ^26^ ^]^ c,d) Characterization of the initial nucleation stage, grown using the conventional process, reveals the typical island growth mode of β‐Ga₂O₃. AFM image of β‐Ga₂O₃ with twins (c) and EBSD map of the (100) surface of β‐Ga₂O₃ with twins (d).

**Figure** **1**a presents the atomic arrangement of the β‐Ga_2_O_3_ unit cell (UC) viewed along the [010] axis, highlighting the two distinct surface terminations, (100) A and (100) B.^[^ ^20^ ^]^ Previous studies have shown that the (100) B termination exhibits a lower surface energy compared to the (100) A termination, which accounts for the domination of (100) B terminations on epitaxial surfaces.^[^ ^22^ ^]^ Two primary types of TBs are observed: TB_(100)_ and TB_(‐102)_. TB_(100)_ B results from a switch in the crystal orientation at the (100) plane, while TB_(‐102)_ forms due to the coalescence of regions with differing twin orientations. The relatively low formation energies of TB_(100)_ B and TB_(‐102)_ as 0.02 and 0.45 J m^−^^2^, respectively, contribute to their high density during the epitaxial growth process (see Figure S1, Supporting Information for further details on the types of epitaxial defects).^[^ ^20^ ^]^

Figure 1b compares the surface energies of the β‐Ga₂O₃ (100) plane with those of other materials, spanning from 2D to 3D structures. Covalently bonded 3D materials, such as Si, Al₂O₃, and GaN, exhibit high surface energies exceeding 1000 mJ m^−^^2^, with GaN reaching values above 2000 mJ m^−^^2^.^[^ ^23^, ^24^, ^25^, ^26^ ^]^ In contrast, the β‐Ga₂O₃ (010) plane has a surface energy of ≈1850 mJ m^−^^2^.^[^ ^14^ ^]^ For 2D materials, where van der Waals forces dominate, surface energies are significantly lower, often below 300 mJ m^−^^2^. For example, graphene and MoS₂ exhibit surface energies below 50 mJ m^−^^2^,^[^ ^16^, ^27^ ^]^ while graphite has a slightly higher surface energy of 228 mJ m^−^^2^.^[^ ^15^ ^]^ The surface energy of the β‐Ga₂O₃ (100) B plane, determined to be 340 mJ m^−^^2^, is the lowest among the various β‐Ga₂O₃ planes, and is comparable to that of graphite.^[^ ^14^ ^]^ This suggests that epitaxy on this plane occurs through a growth process similar to van der Waals epitaxy, rather than conventional 3D homoepitaxial growth.^[^ ^28^ ^]^ Specifically, this leads to a 3D island‐like growth mode, akin to the van der Waals epitaxy of 3D materials on van der Waals surfaces.^[^ ^29^, ^30^, ^31^, ^32^ ^]^

The formation characteristics of twins are observed during the nucleation stage of β‐Ga₂O₃ grown on a (100) substrate using molecular beam epitaxy (MBE). A conventional process was employed to initiate nucleation on the (100) substrate prior to coalescence (see Experimental Section). Atomic force microscopy (AFM) imaging of the surface morphology confirms the predominance of an islandic growth mode, with a nucleation height corresponding to multilayer β‐Ga₂O₃ grown on the (100) substrate (Figure 1c).^[^ ^9^ ^]^ An electron backscatter diffraction (EBSD) map further reveals that the epitaxial islands are partially oriented in directions opposite to that of the substrate, indicating the formation of a significant number of twin domains even during the nucleation stage (Figure 1d).^[^ ^10^ ^]^

### 2.2 Growth and Characterization of Single‐Crystalline β‐Ga_2_O_3_ on (100) Substrate

**Figure 2:**
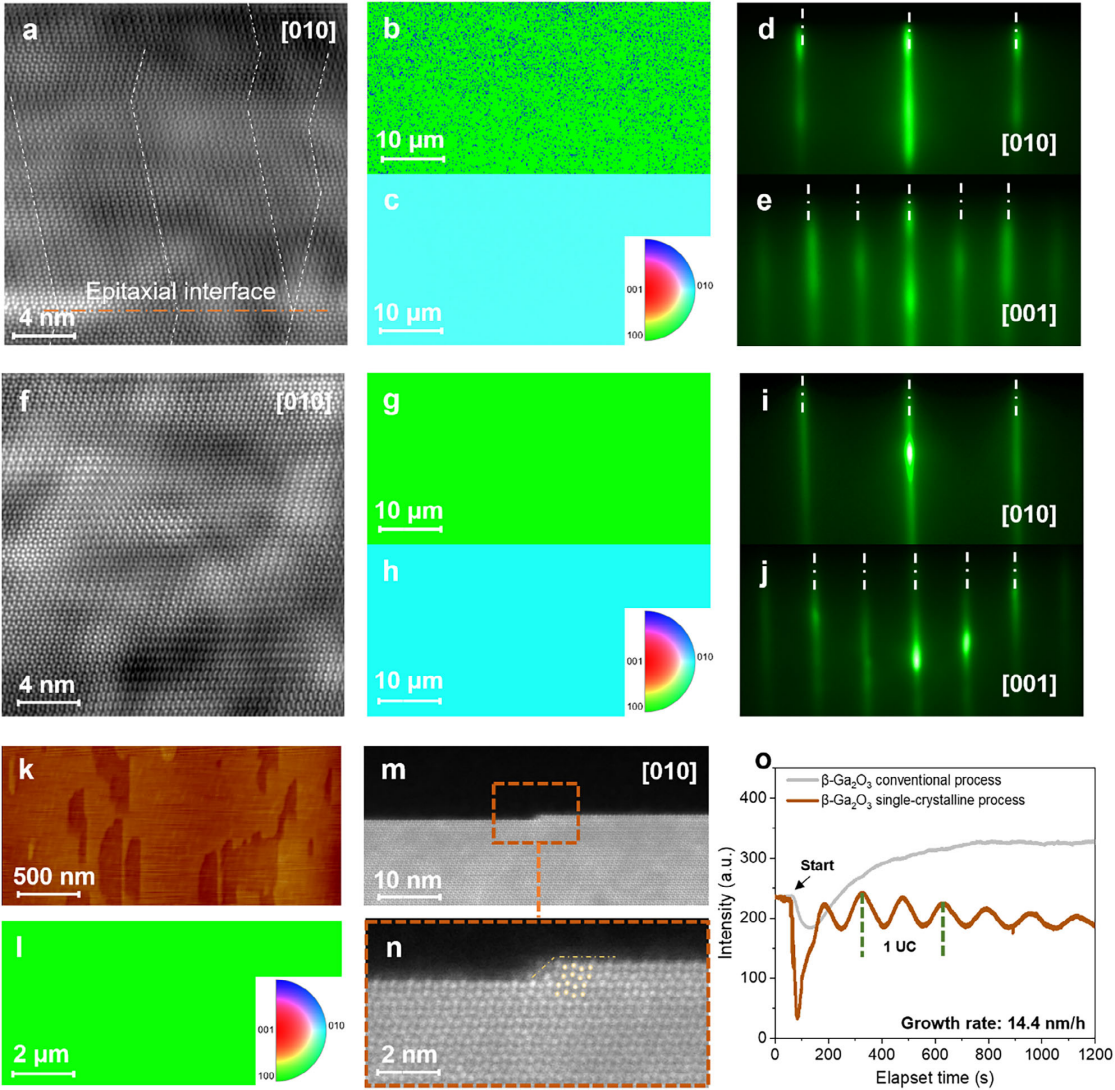
Growth and characterization of polycrystalline and single‐crystalline β‐Ga₂O₃ on a (100) substrate. a) Cross‐sectional HAADF‐STEM image of β‐Ga₂O₃ with twins (conventional process). b,c) EBSD maps of (100) and (010) planes for β‐Ga₂O₃ with twins. d,e) RHEED patterns of β‐Ga₂O₃ with twins, recorded along the [010] and [001] azimuths, respectively. f) Cross‐sectional HAADF‐STEM image of single‐crystalline β‐Ga₂O₃ along the [010] direction. g,h) EBSD maps of (100) and (010) planes for single‐crystalline β‐Ga₂O₃. i,j) RHEED patterns of single‐crystalline β‐Ga₂O₃, recorded along the [010] and [001] azimuths, respectively. k,l) Characterization of the initial nucleation stage for single‐crystalline β‐Ga₂O₃. AFM image showing surface nucleation islands (k) and the corresponding EBSD map (l). m,n) STEM images showing detailed views of a monolayer step of single‐crystalline β‐Ga₂O₃. Monolayer step structure along the [010] direction and a partially enlarged area (n). o) RHEED intensity oscillations for β‐Ga₂O₃ grown using the conventional process and the single‐crystalline process.

As growth progresses, misoriented islands with different orientations coalesce, leading to an increasingly coarse surface due to the formation of extensive twin domains. These defects significantly degrade the crystalline quality (see Figures S2 and S3, Supporting Information).^[^ ^33^ ^]^ The microscopic structure of the twin defects is shown in the cross‐sectional high‐angle annular dark‐field scanning transmission electron microscopy (HAADF‐STEM) image in **Figure** **2**a. This image reveals repeated shifts in the growth orientation along the a‐axis (indicated by the white dashed line), resulting from in‐plane 180° rotations during nucleation.^[^ ^10^ ^]^ The appearance of the TBs at the epitaxial interface (denoted by the red dashed line) confirm that twins form early in the epitaxial process. The detailed formation process of the twin structure is shown in Figure S4 (Supporting Information). Figure 2b,c presents EBSD maps of (100) and (010) plane for the epitaxial β‐Ga₂O₃. The majority of the regions, which are oriented in the same direction as the substrate ((100) plane shown in green, corresponding to the [100] orientation), are interspersed with misoriented domains ((−100) plane shown in blue, corresponding to the [−100] orientation), consistent with the observations in HAADF‐STEM. No directional differences are observed in the [010] orientation, as the twins align with the substrate in this direction (Figure 2c). The twins identified in the EBSD maps arise from in‐plane 180° rotations, driven by the low energy differences between the atomic configurations of the two opposite directions.^[^ ^20^ ^]^ Figure 2d,e shows the reflection high‐energy electron diffraction (RHEED) patterns taken at the end of growth along the [010] and [001] azimuths, respectively. Although the RHEED periodicity in either direction could not distinguish the twin orientation, diffraction streak broadening indicates a rough surface, characteristic of the 3D island growth mode.

To achieve twin‐free single‐crystal β‐Ga₂O₃ epitaxy on a (100) substrate, utilized In as a surfactant and metal‐rich conditions at an elevated growth temperature, facilitating growth approaching thermal equilibrium (see Experimental Section). The use of surfactants is a widely adopted strategy in epitaxial systems to alter the epitaxial kinetics, for example increasing atomic migration distances, or reducing step‐edge barriers.^[^ ^34^, ^35^ ^]^ HAADF‐STEM images show the elimination of twin defects in the β‐Ga₂O₃ films, rendering the interface between the epitaxial layer and substrate indistinguishable (Figure 2f). The absence of TBs and the presence of completely single‐oriented lattices in the epitaxial thin films confirm the high quality and twin‐free structure of the β‐Ga₂O₃ (100) epitaxial layer. These results are further supported by EBSD mappings (Figure 2g,h) and narrow RHEED streaks (Figure 2i,j).

This growth mode effectively eliminates twin misorientation during the nucleation stage. The surface morphology of the nuclei and corresponding EBSD images before coalescence are shown in Figure 2k,l. The EBSD patterns reveal perfectly aligned nuclei, indicating the absence of misorientation. Additionally, the growth mode shifts from 3D island growth to 2D growth mode. This transition is microscopically evident in the HAADF‐STEM images (Figure 2m,n), which show an atomically smooth surface with a half‐layer step edge. Figure 2n provides a magnified view of this step, with the step height corresponding to 1/2 UC and terminating at the (100) B surface. Since the step edges possess higher energy than the surface, the adatoms preferentially incorporate at the half‐layer step edges to minimize the surface energy, extending the step to merge along the (100) B surface in a half‐layer‐by‐half‐layer growth mode. These growth dynamics ensure that the lateral expansion follows the same crystalline orientation as the single‐crystalline nuclei until convergence (Figure S5, Supporting Information). The RHEED oscillation curve (Figure 2o) further supports this lateral growth on a macroscopic scale, with peak intervals corresponding to the growth of a 1/2 UC thickness. In contrast, traditional twin growth conditions do not exhibit oscillation curves due to the 3D island growth mode. By utilizing single‐oriented nucleation and the half‐layer‐by‐half‐layer growth and stitching process, we successfully achieved an atomically flat, single‐crystalline β‐Ga₂O₃ (100) epitaxial layer. Additionally, we utilized X‐ray diffraction (XRD) to macroscopically evaluate the crystal quality of the single‐crystalline film. The rocking curve of the (400) peak exhibited a full width at half maximum (FWHM) of 43.2 arcseconds (Figure S6, Supporting Information). The absence of peak broadening during epitaxial growth confirms the high single‐crystalline quality of the film. Moreover, the 2θ‐ω scan of films grown via the conventional process exhibited interference fringes, which we attribute to interface twinning (Figure S7 and Note S1, Supporting Information). In contrast, films grown via the single‐crystalline process eliminated this phenomenon, indicating improved crystalline quality.

Twins severely degrade the electrical properties of β‐Ga₂O₃, ultimately impacting device performance.^[^ ^36^, ^37^ ^]^ In the thin films grown using our single‐crystalline process, twinning is effectively suppressed, which is expected to significantly enhance electrical performance.

### 2.3 Growth Dynamics of Defect‐Free β‐Ga_2_O_3_

The realization of single‐crystalline epitaxy can be attributed to two critical factors: the formation of single oriented crystal nuclei and the preferential lateral expansion of the nuclei following the orientation of the single‐crystalline nuclei. These requirements are met through the deviation of the conventional growth regime toward thermal equilibrium conditions.

**Figure 3:**
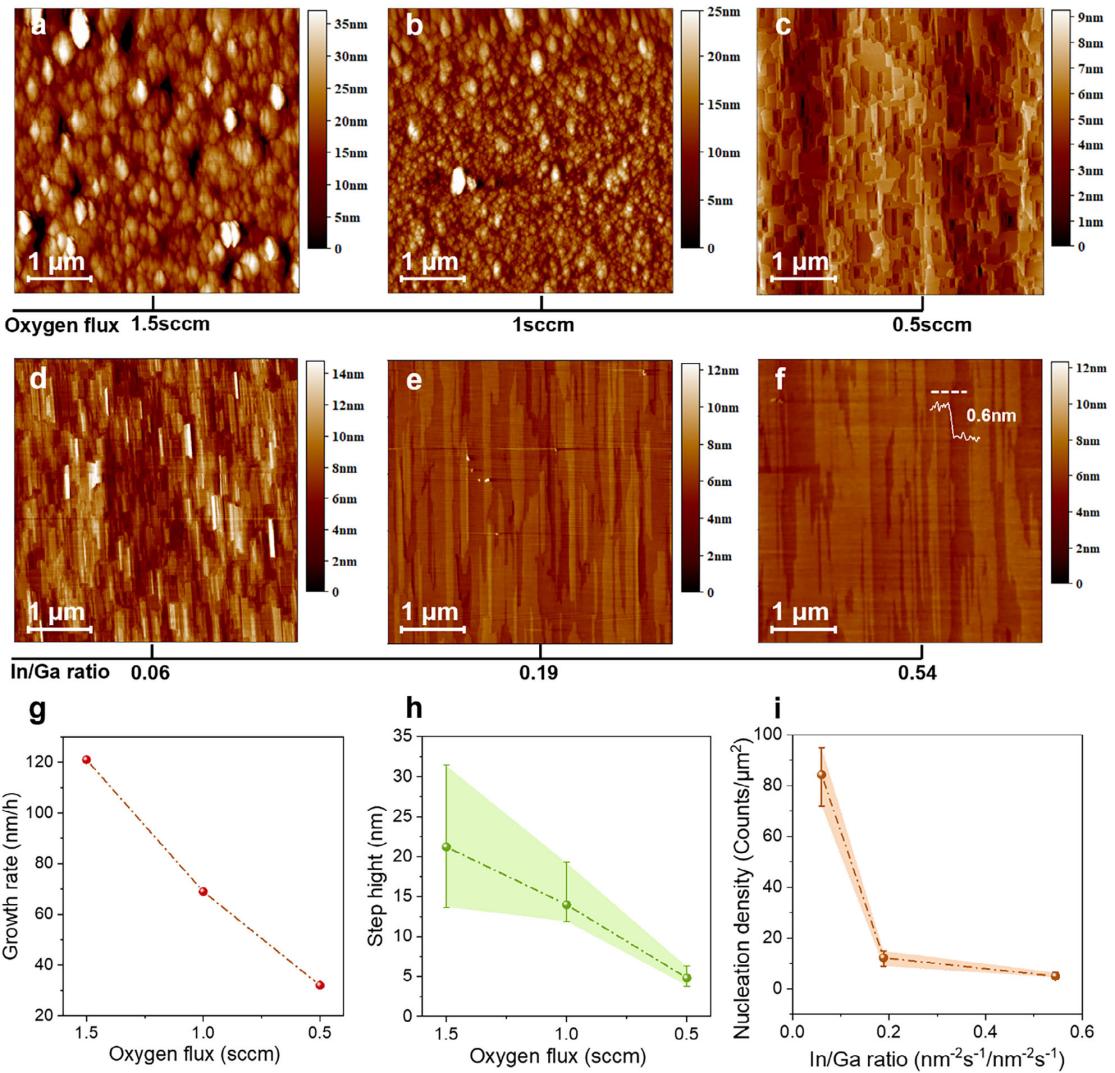
Growth dynamics of single‐crystalline β‐Ga₂O₃. a‐c) AFM images of sample surfaces grown with a fixed Ga K‐cell flux of 2.22 nm⁻^2^ s⁻¹, an In K‐cell flux of 0.05 nm⁻^2^ s⁻¹, and a growth temperature of 800 °C, while progressively decreasing the oxygen flux from 1.5 to 0.5 sccm. d–f) AFM images of sample surfaces grown with a fixed Ga K‐cell flux of 2.22 nm⁻^2^ s⁻¹, an oxygen flux of 0.5 sccm, and a growth temperature of 800 °C, while increasing the In/Ga ratio from 0.06 to 0.54. g,h) Changes in the β‐Ga₂O₃ growth rate (g) and surface step height (h) as a function of oxygen flux, derived from the data in (a–c). i) Nucleation density as a function of the In/Ga ratio, derived from the data in (d–f).

The relatively low formation energy of misoriented twins in the conventional kinetic growth facilitates their formation.^[^ ^20^ ^]^ To counter this, preferential decomposition of misoriented nuclei must be encouraged. First, higher growth temperatures serve as an energy‐driven selection, favoring the decomposition of misoriented twins. However, high growth temperature often destabilizes the growth process, as most atoms are desorbed from the surface and cannot participate in the growth. To overcome this limitation, we introduced an In metal surfactant, which allowed an increase in the growth temperature from 650 to 800 °C. The introduced In preferentially reacts with O to form indium oxides, which exhibit a lower saturated vapor pressure at high temperatures compared to gallium oxides, making them less susceptible to decomposition.^[^ ^38^, ^39^ ^]^ Subsequently, In atoms are replaced by Ga, enabling the growth of β‐Ga_2_O_3_ under elevated temperature conditions.^[^ ^40^, ^41^ ^]^ Second, we reduced the O₂ plasma flow to limit the supply of oxygen to the substrate surface, thereby establishing a metal‐rich growth with high Ga/O ratio. Under high Ga/O ratio conditions, excess Ga reacted with the epitaxial β‐Ga_2_O_3_ to form volatile Ga_2_O,^[^ ^42^ ^]^ which evaporated and provided an additional pathway for the decomposition of misoriented nuclei (**Figure** **3**a–c). For details on the single crystal growth and twin decomposition process of β‐Ga₂O₃, see Note S2 and Figures S10 and S11 (Supporting Information). The preferential decomposition of misoriented twin nuclei was accompanied by a significant reduction in the epitaxial growth rate (Figure 3g) and a decrease in step height (Figure 3h). AFM results provided direct evidence of this transition, showing a change in surface morphology from rounded islands to rectangular‐shaped surfaces aligned with the equilibrium shape of monoclinic configuration (Figure 3a–c). This indicated a growth process shifted toward the thermal equilibrium regime. Therefore, single‐crystalline nucleation was successfully achieved on the β‐Ga_2_O_3_ (100) surface, confirmed by EBSD mapping of initial nuclei as shown in Figure 2k,l.

Enhanced adatom surface mobility is critical for Ga adatoms to migrate across the surface and incorporate at energetically favorable step edges, facilitating a half‐layer‐by‐half‐layer growth mode. By reducing the oxygen supply and increasing the In/Ga ratio, we also achieved a significant enhancement in Ga atom diffusion, as evidenced by a decrease in nucleation density from ≈84.25 to 5 nuclei µm^−2^ (Figure 3i). Using mean‐field nucleation theory,^[^ ^43^, ^44^ ^]^ we performed a semi‐quantitative analysis of the Ga adatom diffusion barrier and diffusion distance (details in Note S3, Supporting Information).^[^ ^45^, ^46^, ^47^, ^48^, ^49^ ^]^ The diffusion barrier of Ga adatoms (E_d_) decreased from 1.33 to 0.45 eV, while the diffusion constant (D) increased from 2.45 × 10^−10^ to 1.8 × 10^−6^ cm^2^ s^−1^ (Table S1, Supporting Information). This diffusion constant is three orders of magnitude higher than the previously reported value for Ga atoms without an In surfactant (7 × 10^−9^ cm^2^ s^−1^),^[^ ^10^ ^]^ and is comparable to diffusion constants for metal atoms on 2D material surfaces (Table S2, Supporting Information).^[^ ^49^ ^]^ The increased migration distance of Ga adatoms supports the preferential lateral film expansion along step edges,^[^ ^50^ ^]^ enabling their coalescence into a continuous film in a half‐layer‐by‐half‐layer growth mode. This is also evidenced by a reduction in step height, as confirmed by AFM measurements showing a step height decreased from 3.89 to 0.6 nm (Figure 3d–f) and corroborated by RHEED oscillation curves (Figure 2o). The growth mode transition is accompanied by a gradual decrease of twins density (Figure S12, Supporting Information).

### 2.4 Growth Phase Diagram of β‐Ga_2_O_3_ on (100) Surface

**Figure 4:**
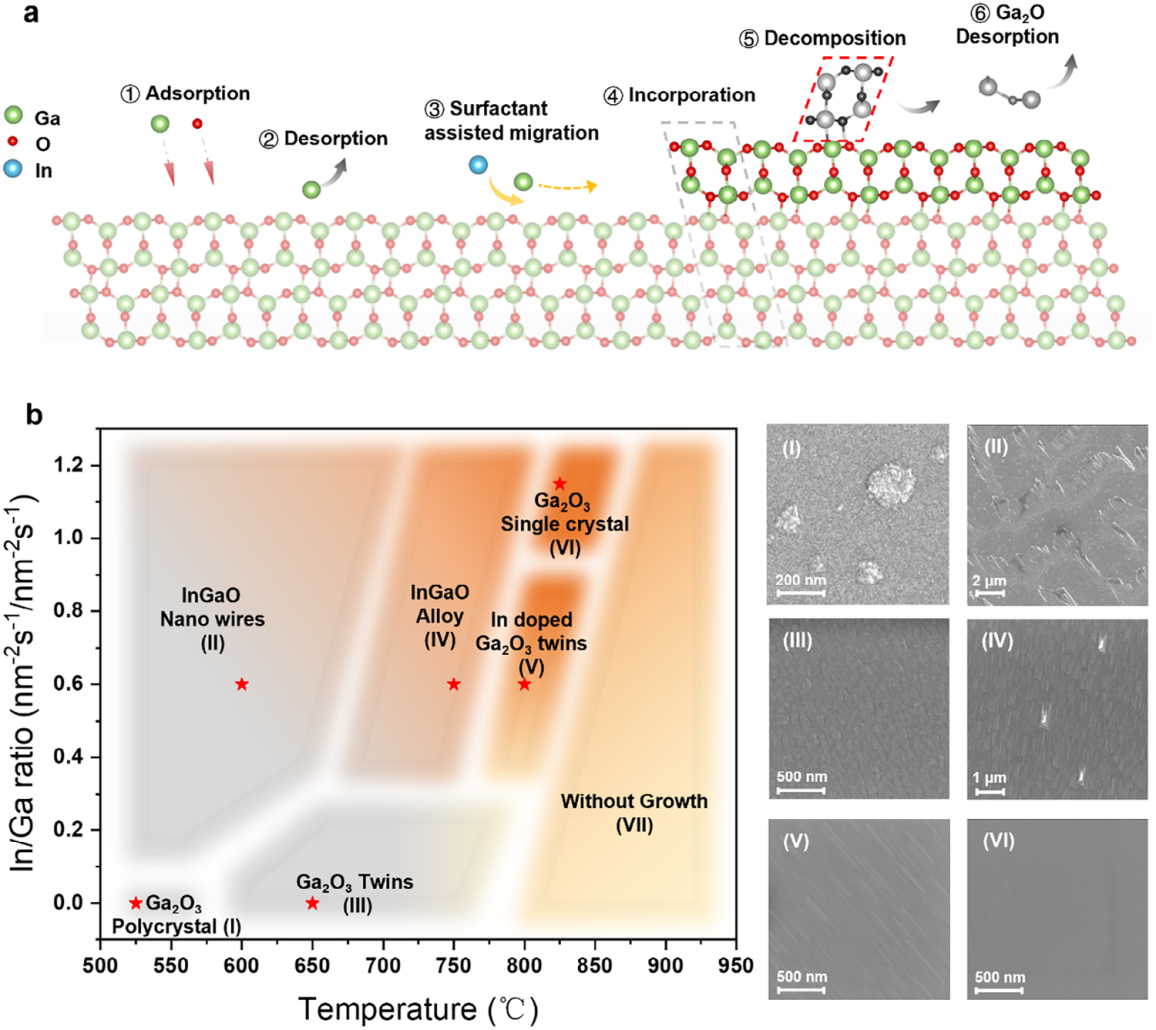
Schematics and phase diagram for single‐crystalline homoepitaxy of β‐Ga₂O₃ on (100) substrates. a) Schematic illustration of the growth process for single‐crystalline β‐Ga₂O₃ with In surfactant. b) Phase diagram for β‐Ga₂O₃ growth with In surfactant, plotted against growth temperature and In/Ga ratio. The diagram includes corresponding SEM images of surface morphologies for each identified growth regime (I‐VI).

The schematics illustrating the homoepitaxial growth of single‐crystalline β‐Ga₂O₃ on the (100) surface are presented in **Figure** **4**a, detailing the sequential key steps of the process.^[^ ^51^ ^]^ During the initial stages (Steps 1 and 2), Ga, In, and O atoms are introduced to the substrate surface, where they adsorb. Due to the elevated growth temperatures, some adatoms undergo partial desorption. Steps 3 and 4 highlight the diffusion of Ga adatoms, facilitated by the presence of the In surfactant, which enhances Ga migration toward step edges, promoting incorporation and lateral layer expansion (Figure 3d–f). Steps 5 and 6 describe decomposition events during growth (Figure 3a–c), wherein high growth temperatures and an excess of Ga adatoms lead to the formation of volatile suboxide Ga₂O, facilitating the decomposition of twin structures.

Figure 4b presents a phase diagram for β‐Ga₂O₃ growth, illustrating the dependence of the crystal growth regime on temperature and the In/Ga ratio. Surface morphologies corresponding to six distinct growth regions are captured in SEM images. In region I, where no In flux is applied and the growth temperature is below 600 °C, polycrystalline films are observed, characterized by large surface islands (Figure S13, Supporting Information). In region II, introducing In flux at these temperatures results in the formation of InGaO nanowires due to insufficient energy for In desorption, allowing In to catalyze nanowire growth (Figure S14, Supporting Information). As the growth temperature increases, SEM images in region III reveal the formation of 3D islands, indicative of β‐Ga₂O₃ twins. In region IV, further increases in the In/Ga flux ratio and temperature result in a rectangular surface morphology, indicating In incorporation and a phase transition to cubic InGaO alloys (Figure S15, Supporting Information).^[^ ^52^ ^]^ With additional temperature increases, nucleation streaks appear, corresponding to In‐doped β‐Ga_2_O_3_ twins in region V. Finally, in region VI, a higher In/Ga ratio leads to a twin‐free single‐crystal phase due to optimized van der Waals epitaxial growth mode. Increasing the growth temperature beyond this range would enter region VII, where complete decomposition prevents film growth.

## 3 Conclusion

In this study, we successfully demonstrated the homoepitaxial growth of single‐crystalline β‐Ga₂O₃ on an on‐axis (100) substrate using a van der Waals epitaxial strategy, akin to approaches employed for single‐crystalline 2D material growth. By optimizing growth conditions with elevated temperatures and a high Ga/O ratio, we established a growth regime that enabled single‐oriented nucleation on step‐free surfaces. The incorporation of a metallic In surfactant further enhanced Ga adatom mobility, increasing the diffusion constant by three orders of magnitude compared to conventional twins growth conditions. This enhancement facilitated the migration and incorporation of Ga adatoms at single‐crystalline nucleation step edges, enabling the coalescence of a continuous film via a half‐layer‐by‐half‐layer growth mechanism.

This approach deviating from conventional growth regimes, approximates thermal equilibrium conditions, eliminating twin grain boundaries, and achieving atomically flat surfaces. Leveraging the advantageous material properties of β‐Ga₂O₃ and the availability of large‐diameter single‐crystalline substrates, our findings open new opportunities for the development of high‐performance and cost‐effective β‐Ga₂O₃ based electronic devices. Importantly, this work establishes the viability of scalable epitaxial growth on (100) surfaces, which has previously been constrained by the prevalence of twin defect formation.

## 4 Experimental Section

### Materials Growth

The β‐Ga_2_O_3_ thin films were epitaxially grown using a plasma‐assisted molecular beam epitaxy (PA‐MBE) system. This homebuilt MBE system is equipped with Ga and In shuttered K‐cells as metal sources, a radio‐frequency plasma source to provide activated oxygen, an ion gauge to measure beam‐equivalent pressures (BEPs), and an in situ RHEED detection system.

The epitaxial substrates were unintentionally doped (UID) β‐Ga_2_O_3_ (100) substrates. Prior to epitaxial growth, the substrates underwent a standard organic cleaning process, including ultrasonic cleaning in acetone, isopropanol, and deionized water for 5 min each. After cleaning, the substrates were dried using a nitrogen gun for 15 min. The cleaned substrates were then attached to a 2‐inch silicon wafer using In bonding and loaded into the transfer chamber, where they were baked at 250 °C for 2.5 h. Following this, they were transferred to the growth chamber, where they were maintained at 850 °C for 30 min to decompose the surface amorphous layer. Subsequently, the heating stage was cooled to a lower temperature for growth. During the growth process, a RHEED system was employed to monitor the epitaxial quality and thickness in real time.

The growth process with twin β‐Ga_2_O_3_ was operated at a growth temperature of 650 °C, with the Ga K‐cell heated to 750 °C at a ramp rate of 10 °C min^−1^ (Ga flux of 1.81 nm^−2^ s^−1^), and the O₂ plasma maintained at 250 W with a flow rate of 1.5 sccm. To characterize the initial nucleation stage, the sample was grown for 5 min.

For the single‐crystalline β‐Ga_2_O_3_ growth condition, the growth temperature was set to 800 °C, with the O₂ plasma maintained at 250 W and a flow rate of 0.5 sccm. The Ga and In K‐cells were heated to 800 and 700 °C, respectively, with a ramp rate of 10 °C min⁻¹, resulting in Ga and In fluxes of 2.22 and 1.2 nm^−2^ s^−1^, respectively. To characterize the initial nucleation stage and the morphology of the nuclei, the sample was grown for 10 min.

To investigate the evolution of surface morphologies and growth regimes, the growth parameters were modulated by varying the oxygen flux and the In‐to‐Ga flux ratio. The growth temperature was maintained at 800 °C with a fixed Ga flux of 2.22 nm^−2^ s^−1^. In Figure 3a–c, the In flux was set to 0.05 nm^−2^ s^−1^, while the O₂ flow rates were adjusted to 1.5, 1.0, and 0.5 sccm, respectively. In Figure 3d–f, the O₂ flow rate remained constant at 0.5 sccm, and the In flux was varied to 0.14, 0.42, and 1.2 nm^−2^ s^−1^, respectively.

### Atomic Force Microscopy

An AFM (Dimension ICON, Bruker) was used to characterize the surface morphology and thickness of the steps, employing non‐metal‐coated probes (RTESP‐300, Bruker) in standard tapping mode.

### Scan Electron Microscopy

A SEM (Gemini500, Zeiss) was used to characterize the surface morphology of the β‐Ga_2_O_3_ films at an accelerating voltage of 5 kV. Energy‐dispersive X‐ray spectroscopy (EDS) elemental maps were conducted by the EDS detector (Oxford Instruments) equipped in SEM system. An EBSD detector (Oxford Instruments) within the SEM enabled the acquisition of electron backscatter patterns, providing information on the crystal structure and film quality.

### X‐Ray Photoelectron Spectroscopy

An XPS (ESCALAB Xi+, Thermo Fisher) was used to analyze the surface and depth composition of the films, with Ar ion sputtering applied for depth profiling. AFM was then employed to measure the etch pit depth (0–13 nm) resulting from sputtering.

### X‐Ray Diffraction

An XRD (D8 Bruker) was used to measure 2θ‐ω scan and rocking curve with a Cu 471 target.

### High‐Angle Annular Dark‐Field Scanning Transmission Electron Microscopy

HAADF‐STEM images were obtained using a spherical aberration‐corrected transmission electron microscope (Thermo Fisher Scientific Spectra Ultra) operating at 300 kV. The probe convergence semi‐angles were set to 15 and 21 mrad, while the annular detector collection angle ranged from 24 to 121 mrad and from 69 to 200 mrad, respectively. All low‐order aberrations were corrected to acceptable levels, achieving a spatial resolution of ≈0.75 Å. The cross‐sectional TEM sample was prepared using FIB milling with a Thermo Fisher Scientific Helios 5 UX system. Carbon (C) and platinum (Pt) were sequentially deposited on the sample surface to protect the films during the preparation process.

## Conflict of Interest

The authors declare no conflict of interest.

## Author Contributions

T.J. and H.W. contributed equally to this work. Material growth was performed by T.J., H.W., J.L., and Y.M. SEM and EBSD were performed by H.W. and Y.M. AFM and XPS were performed by H.W. and J.C. Electron microscopy was performed by H.Z., T.J., and Z.Y. β‐Ga_2_O_3_ substrates were provided by X.H. and S.Z. The manuscript was co‐written by T.J., H.W., and W.K. The research was supervised by W.K. All authors contributed to discussions and commented on the manuscript.

## Supporting information



Supporting Information

Supplemental Tables

## Data Availability

The data that support the findings of this study are available from the corresponding author upon reasonable request.
