# Supplementary material for: Single‐Crystalline β‐Ga2O3 Homoepitaxy on a Near Van der Waals Surface of (100) Substrate
Source: Adv Sci (Weinh). 2025 Mar 8;12(17):2417436. doi: 10.1002/advs.202417436 (PMC12061327; doi:10.1002/advs.202417436)
Supplement: Supplementary file 1 — Supporting Information [file ADVS-12-2417436-s002.docx]

**Supporting Information**

**Single-Crystalline β-Ga_2_O_3_ Homoepitaxy on a Near Van der Waals Surface of (100) Substrate**

*Tong Jiang, Hao Wang, Huaze Zhu, Junwei Cao, Xiaoqing Huo, Zhiqing Yang, Junshuai Li, Yaqing Ma, Shengnan Zhang, Xiang Xu, Wei Kong**

**Contents**

**Note S1:** XRD characterization of β-Ga₂O₃ (100) thin films grown via conventional process and single-crystalline process

**Note S2:** The growth and decomposition process of β-Ga₂O₃

**Note S3:** Calculation of diffusion constants

**Figure S1:** Schematic side views of two types of twin boundaries and stacking faults for β-Ga_2_O_3_ (100) plane and their formation energy.

**Figure S2:** Epitaxial films with twin defects grown by conventional methods.

**Figure S3:** Surface morphologies of epitaxial films grown by conventional methods.

**Figure S4:** The flow diagram of epitaxial growth for β-Ga₂O₃ with twins.

**Figure S5:** Surface morphology and EBSD analysis of samples grown under the single-crystalline process at different growth durations.

**Figure S6:** XRD characterization of the epitaxial β-Ga_2_O_3_ (100) thin film grown via a single-crystalline process.

**Figure S7:** XRD comparison of β-Ga₂O₃ (100) thin films grown via conventional process and single-crystalline process.

**Figure S8:** The XRD characterization of heteroepitaxial β-Ga_2_O_3_ films on sapphire substrates.

**Figure S9:** The SEM and AFM morphological images of heteroepitaxial β-Ga_2_O_3_ films on sapphire substrates.

**Figure S10:** The growth scheme for Ga_2_O_3_ and the decomposition of misoriented Ga_2_O_3_ nuclei.

**Figure S11:** The surface morphologies of the β-Ga_2_O_3_ substrate before and after Ga etching.

**Figure S12:** EBSD images of epitaxial films under growth conditions where the In/Ga ratio increased from 0.06 to 0.54.

**Figure S13:** EBSD images of epitaxial polycrystalline β-Ga_2_O_3_ at growth temperature below 600°C.

**Figure S14:** SEM and EDS images of the InGaO nanowires.

**Figure S15:** SEM and EDS images of the InGaO alloyed thin film.

**Figure S16:** Depth profiling XPS analysis of single-crystal epitaxial (100) β-Ga_2_O_3_.

**Table S1:** Calculation of Ga atom migration.

**Table S2:** Comparison of diffusion constants of In and Ga atoms on different material surfaces.

**Note S1:** XRD characterization of β-Ga₂O₃ (100) thin films grown via conventional process and single-crystalline process

We have measured the XRD data for β-Ga₂O₃ (100) thin films, as presented in Figure S7. In the conventional epitaxial process, the 2θ-ω scan exhibits pronounced interference fringes, which arise from severe lattice distortion at the epitaxial interface due to the presence of numerous twin domains, leading to X-ray interference. Similar phenomena have been reported in previous studies. ^[1,2]^
 In contrast, the epitaxial film grown using the single-crystalline process (under metal-rich conditions) does not show interference fringes in its 2θ-ω scan. This indicates that the lattice constant remains unchanged throughout the epitaxial process and that the film maintains the same structural quality as the substrate, further confirming its high single-crystal quality. Notably, the rocking curve of the (400) peak exhibits a full width at half maximum (FWHM) of 43.2 arcseconds (Figure S6), comparable to that of the high-quality single-crystal substrate used.

**Note S2:** The growth and decomposition process of β-Ga₂O₃

For the β-Ga₂O₃ growth, thermodynamic calculations and experimental results demonstrate that gallium and oxygen initially form the suboxide Ga₂O (gas). This Ga₂O then reacts with the remaining oxygen atoms to form Ga₂O₃ (solid). The reaction path described below has been widely reported in the previous studies: ^[1,3]^

2Ga(a) + O(a) $\longrightarrow$ Ga_2_O(g), (1)

Ga2O(g) + 2O(a) $\longrightarrow$ Ga_2_O_3_(s), (2)

with (a), (g), and (s) denoting the adsorbed, gaseous, and solid phase, respectively.

Under gallium-rich at high-temperature conditions, gallium oxide can react with excess gallium to form Ga₂O (solid-to-gas transition), leading to the preferential etching of gallium metal. The chemical reaction is:

4Ga (a)+ Ga_2_O_3_(s) $\longrightarrow$ 3Ga_2_O(g). (3)

The entire process is illustrated in the flowchart in Figure S10. In our study, the single-crystalline growth of β-Ga₂O₃ (100) films occurs under Ga-rich conditions. Under these growth conditions, twin domains, which have relatively higher free energy than single-crystalline domains, are less stable and more prone to reacting with excess Ga. This reaction leads to the formation of gaseous Ga₂O, which subsequently desorbs from the surface. Furthermore, the high growth temperature (800°C) acts as a thermodynamic selection, further facilitating this reaction and ultimately promoting the decomposition of twin domains.

**Note S3:** Calculation of diffusion constants

Mean-field nucleation theory has been extensively employed to estimate the relationship between the diffusion constant and nucleation density. Liu et al. demonstrated this approach to semi-quantify Au deposition on 2D materials using the simplified mean-field model. For 2D island growth, numerous studies have been conducted to estimate these parameters. We follow this model to apply it to our near van der Waals epitaxial growth mode of (100) β-Ga₂O₃. Based on these previous studies, ^[4,5]^ we find that the nucleation density ($N$) is proportional to the activation energy for diffusion ($E_{d}$), as: ^[6-8]^

$N\propto exp\left( \frac{i}{i+2}\frac{E_{d}}{kT} \right)$ (1)

where $i$ is the critical nucleus size. Normally, if we treat the growth process in a simplified manner and neglect re-evaporation, for 2D island growth, the critical nucleus $i$ is typically 1 or 2. We choose $i=1$ in our calculations. $E_{d}$ is the activation energy for diffusion, $k$ is the Boltzmann constant, and $T$ is the growth temperature.

The diffusion constant ($D$) can be calculated using the following equation: ^[6]^

$D= \left( a^{2}\nu_{d}/4 \right) exp\left( -\frac{E_{d}}{kT} \right)$ (2)

where $a$ represents the atomic spacing distance. The average atomic spacing, 3.18 Å, as the value for $a$ in the calculations. $\nu_{d}$ is the effective surface vibration frequency (~10¹¹ - 10¹³ s⁻¹). An attempt frequency $\nu_{d}$ = 10^12^ s^-1^ will be applied in the equation.

The diffusion length ($L_{D}$) was proposed by Thomson et al. as a function of nucleation density ($N$), as expressed by the following equation: ^[9]^

$L_{D}= \frac{1}{\sqrt{N}}$ (3)

**
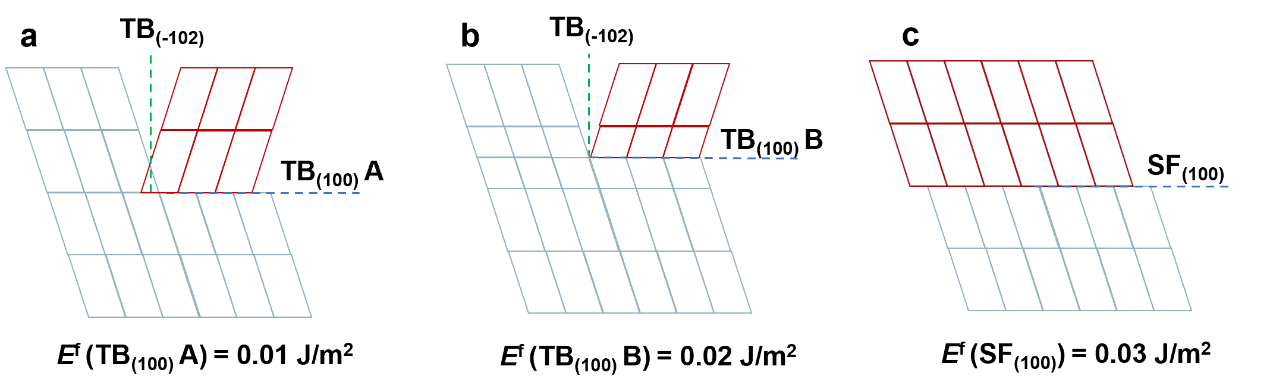
Figure S1:** Schematic side views of two types of twin boundaries and a stacking fault for β-Ga_2_O_3_ (100) plane and their formation energy. a) Stick model of the twin boundaries formed on the (100) A plane of β-Ga_2_O_3_ with the formation energy of E^f^=0.01 J/m^2^. b) Stick model of the TBs formed on the (100) B plane of β-Ga_2_O_3_ with the formation energy of E^f^=0.02 J/m^2^. c) Stick model of the stacking fault formed on the (100) B plane of β-Ga_2_O_3_ with the formation energy of E^f^=0.03 J/m^2^. The termination surface of the β-Ga_2_O_3_ (100) plane is typically the (100) B, and thus, TB_(100)_ B type is commonly observed in conventional epitaxy, despite its formation energy not being the lowest, as shown in Figure 1a. The formation energy of TB_(-102)_ is 0.45 J/m^2^. ^[10]^

**
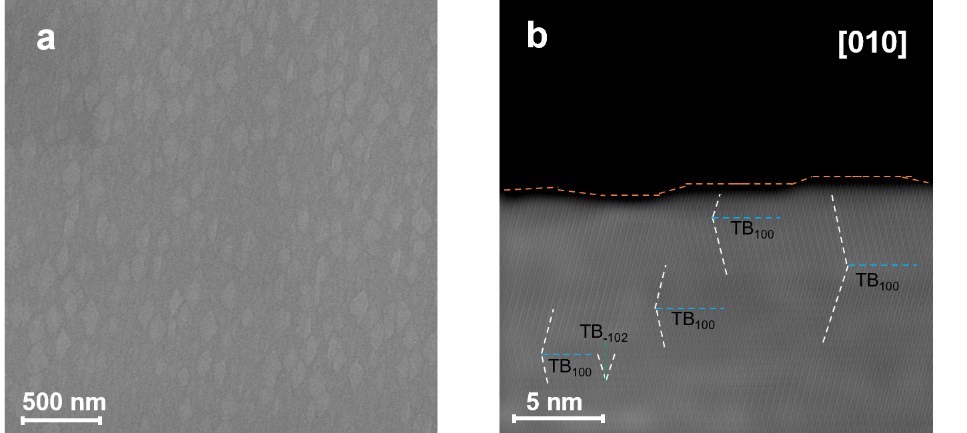
**

**Figure S2:** Epitaxial films with twin defects grown by conventional methods. a) SEM image shows the island surface morphology of the epitaxial film. b) The cross-sectional STEM image reveals the presence of randomly generated twin boundaries: TB_(100)_ parallel to the epitaxial interface and TB_(-102)_ perpendicular to the interface. The blue and green dashed lines represent the TB_(100)_ and TB_(-102)_, respectively, and the white dashed line indicates the a-axis direction of crystal.

**
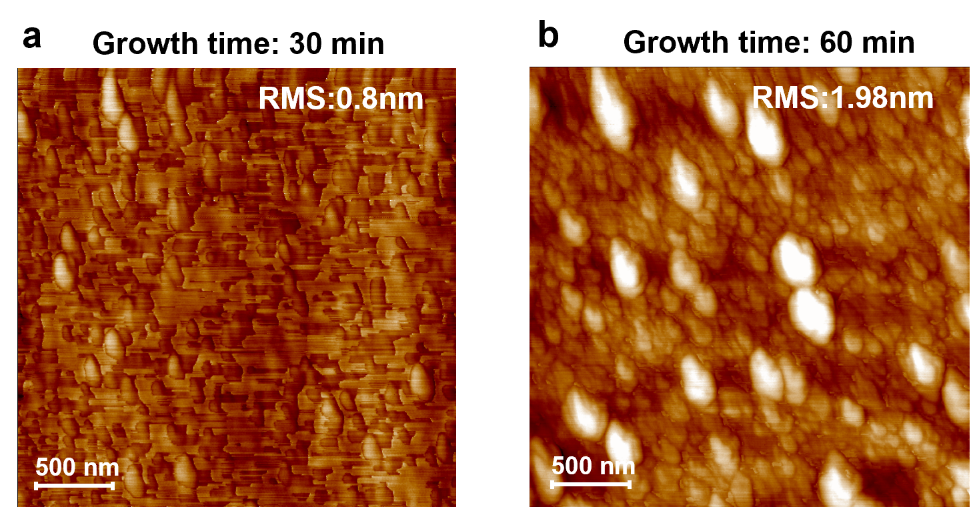
**

**Figure S3:** Surface morphologies of epitaxial films grown by conventional methods**.** a) AFM image of the film surface after 30 minutes of growth, with a roughness of 0.8 nm. b) AFM image of the film surface after 60 minutes of growth, with a roughness of 1.98 nm.


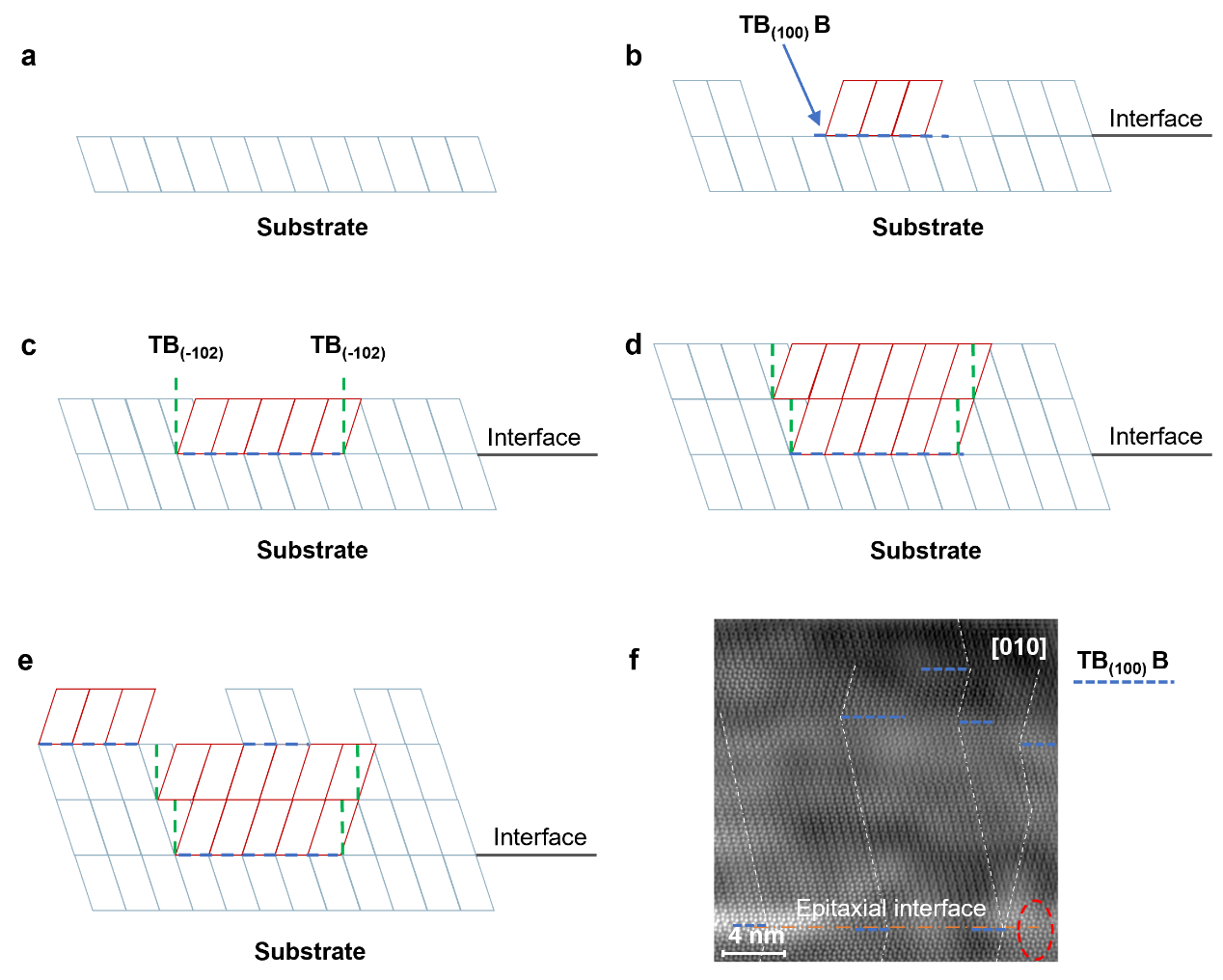


**Figure S4:** The flow diagram of epitaxial growth for β-Ga₂O₃ with twins. a-e) the schematic diagrams of β-Ga₂O₃ epitaxial growth over time. The blue and green dashed line in figure represent the TB_(100)_ B and TB_(-102)_, respectively. f) the corresponding cross-sectional HAADF-STEM image of β-Ga₂O₃ with twins, and the orange and blue dashed line represent the epitaxial interface and TB_(100)_ B. The red circle indicates the single crystal area.


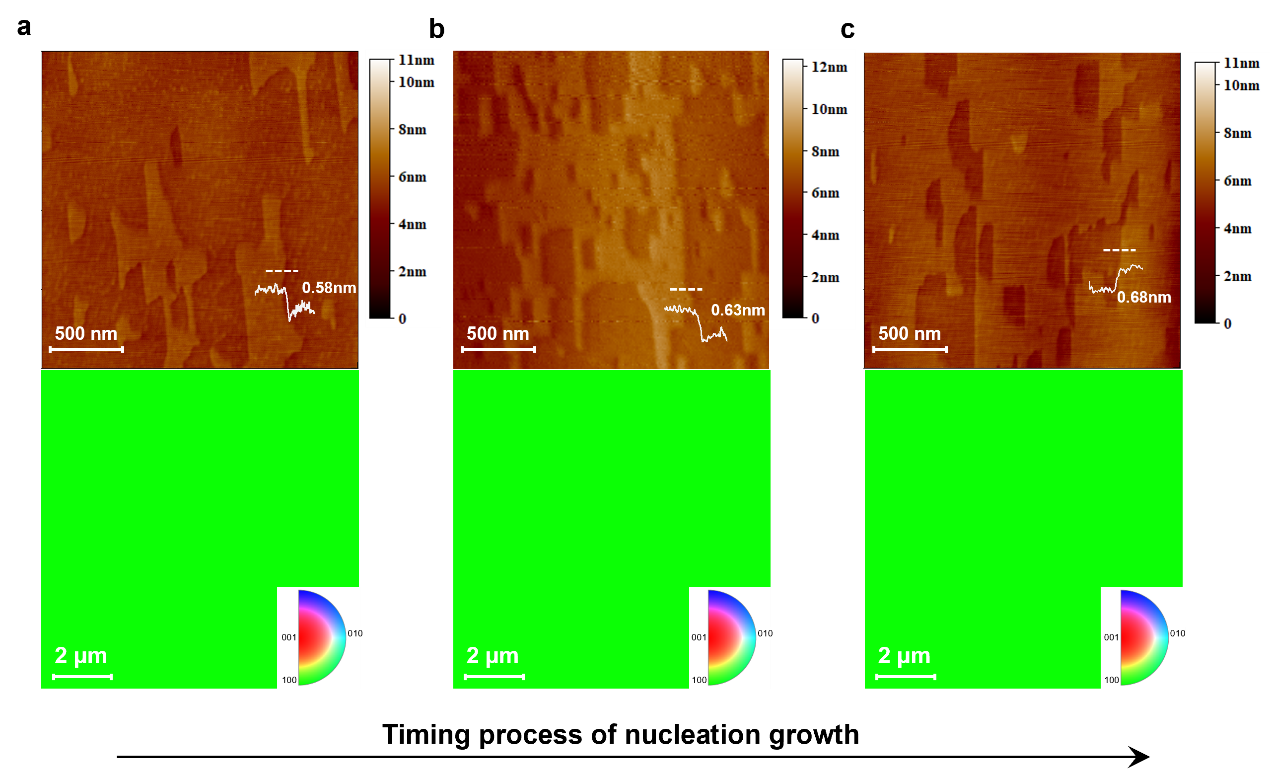


**Figure S5:** Surface morphology and EBSD analysis of samples grown under the single-crystalline process at different growth durations. a-c) The surface gradually transitions from isolated crystal nuclei to a continuous film with the growth time increase.

**
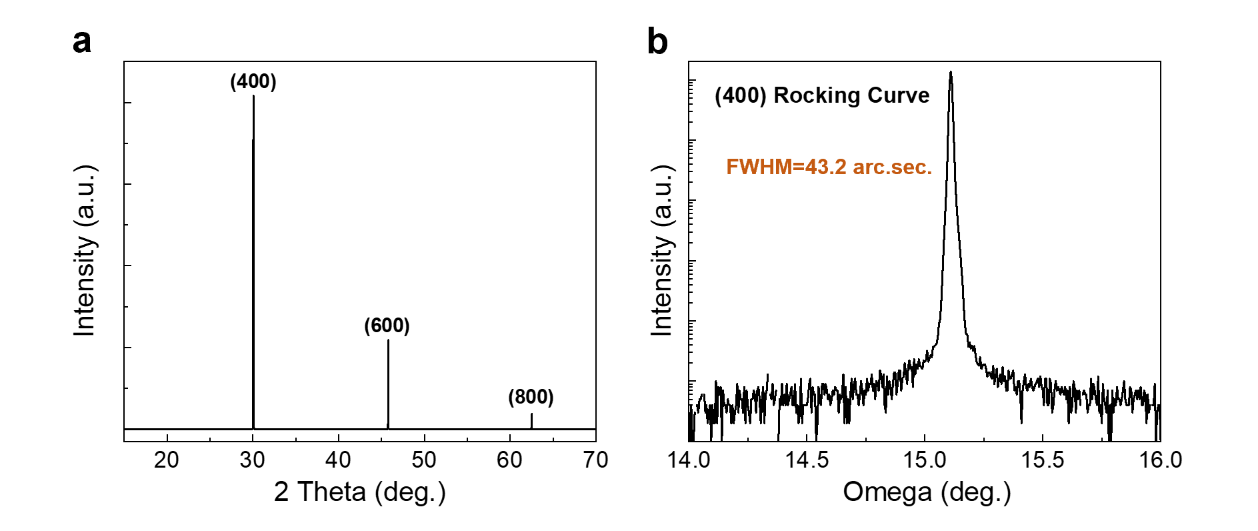
**

**Figure S6:** XRD characterization of the epitaxial β-Ga_2_O_3_(100) thin film (200 nm) grown via a single-crystalline process. a) 2θ-ω scan of the β-Ga_2_O_3_(100) epitaxial thin film, showing peaks corresponding to (400), (600), and (800). b) Rocking curve of the (400) peak of the β-Ga_2_O_3_(100) epitaxial thin film, with a FWHM of 43.2 arcseconds.

**
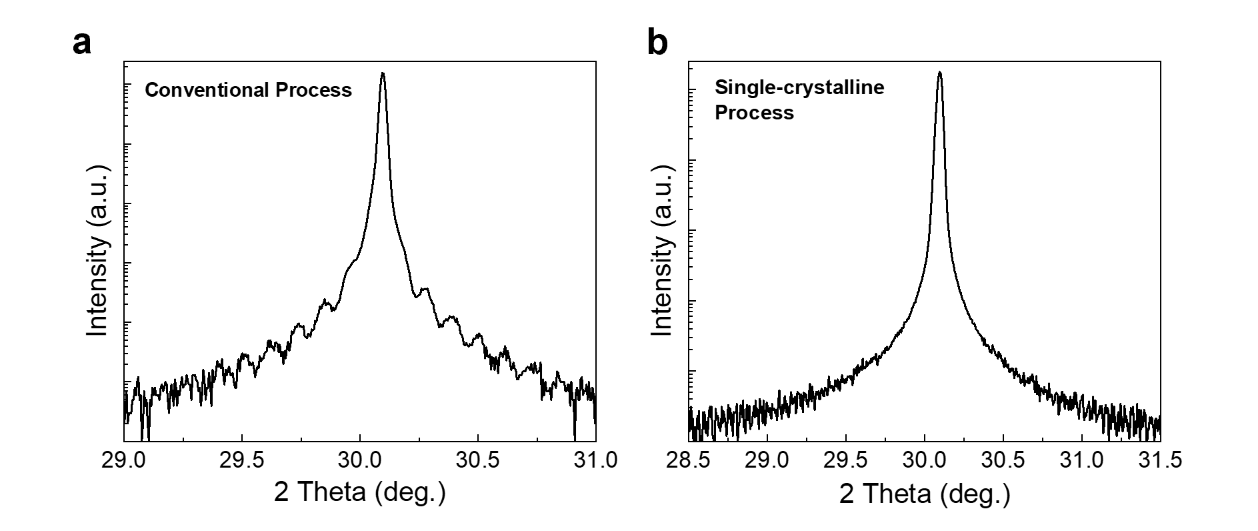
**

**Figure S7:** XRD comparison of β-Ga₂O₃ (100) thin films (80nm) grown via conventional process and single-crystalline process. a) 2θ-ω scan of the (400) peak for the thin films grown via conventional process. b) 2θ-ω scan of the (400) peak for the thin films grown via single-crystalline process.

.

**
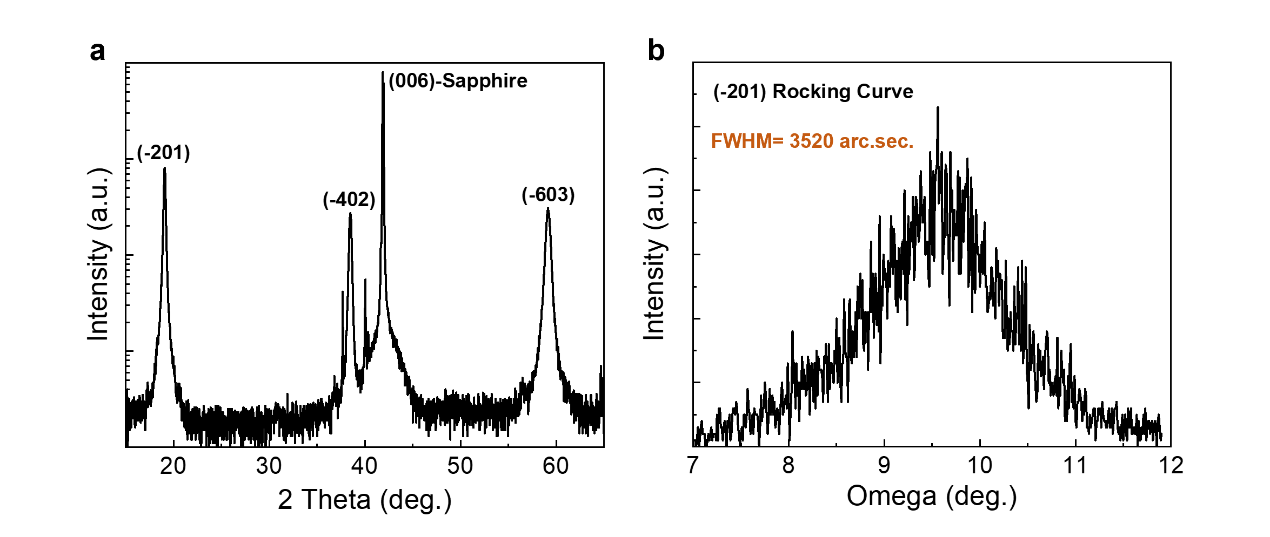
**

**Figure S8:** The XRD characterization of heteroepitaxial β-Ga_2_O_3_ films on sapphire substrates. a) 2θ-ω scan of the β-Ga_2_O_3_ film grown on sapphire, showing β-Ga_2_O_3_ peaks corresponding to (-201), (-402), and (-603) and sapphire peak of (006). b) Rocking curve of the (-201) peak of the β-Ga_2_O_3_ film, with a FWHM of 3520 arcseconds.


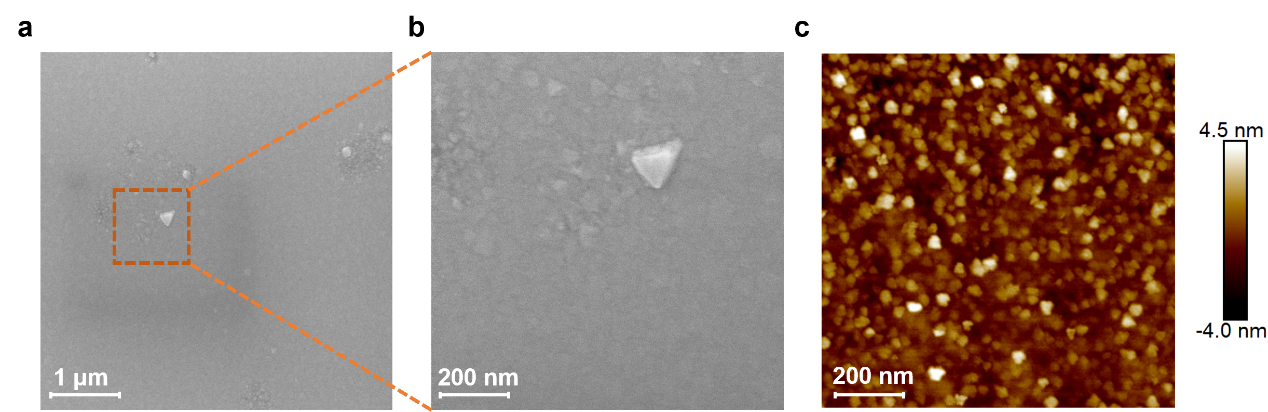


**Figure S9:** The SEM (a, b) and AFM (c) morphological images of heteroepitaxial β-Ga_2_O_3_ films on sapphire substrates.

Compared to other large-scale β-Ga₂O₃ epitaxial growth techniques, such as large-area heteroepitaxial growth on sapphire (Figures S8 and S9), our process offers superior crystal quality. This advantage makes β-Ga₂O₃ (100) thin films highly promising for applications in solar-blind photodetectors and power electronics.

**
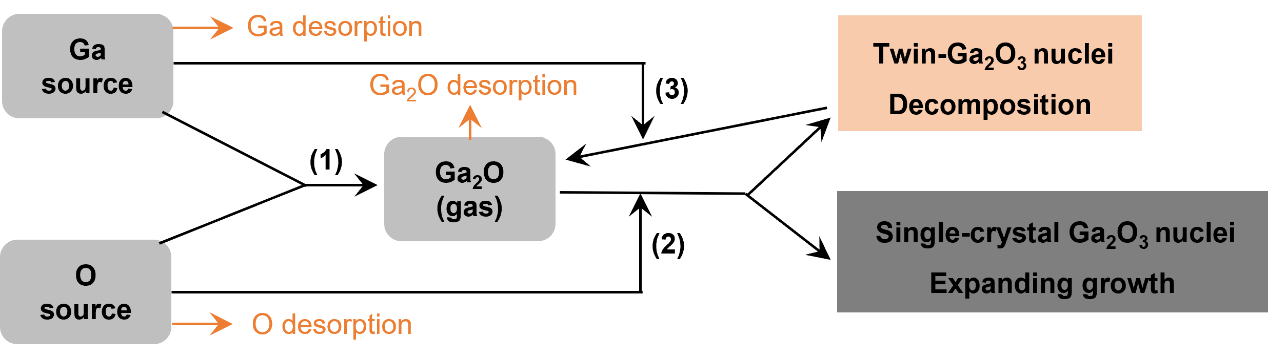
**

**Figure S10.** The growth scheme for Ga_2_O_3_ and the decomposition of misoriented Ga_2_O_3_ nuclei.


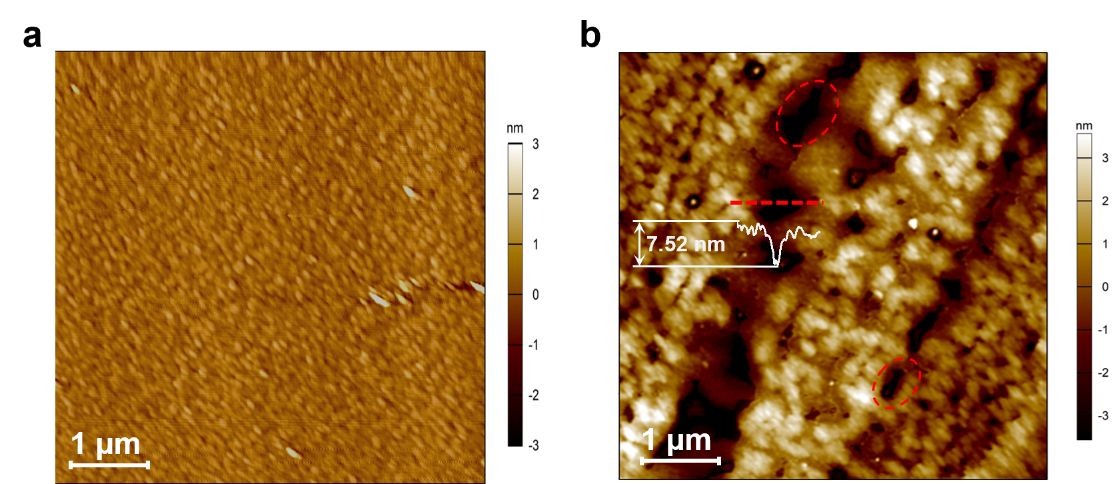


**Figure S11:** The surface morphologies of the β-Ga_2_O_3_ substrate before (a) and after (b) Ga etching. The red circle areas are the Ga etching pits.


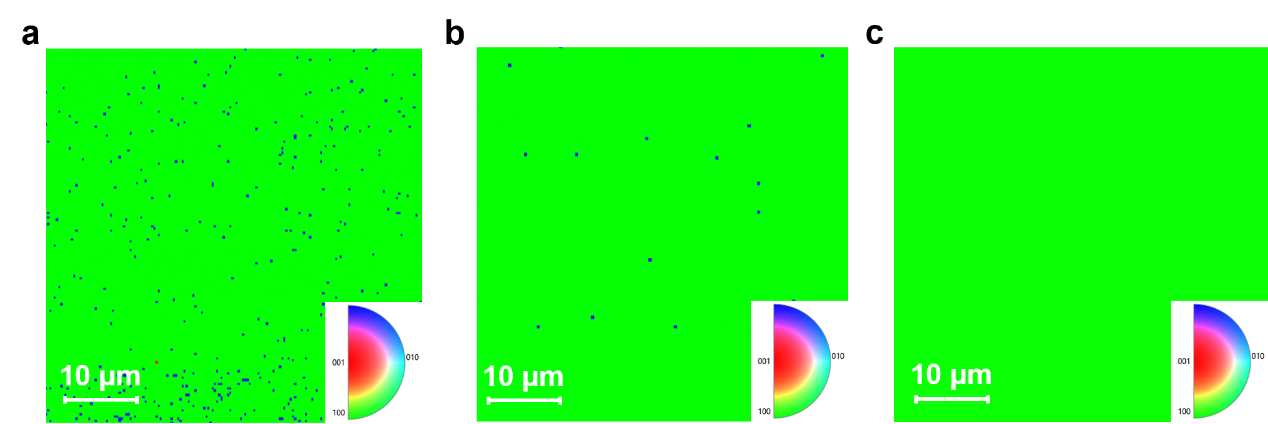


**Figure S12.** EBSD images of epitaxial films. a-c) Growth conditions where the In/Ga ratio increased from 0.06 to 0.54, corresponding to Figure 3d-f.

**
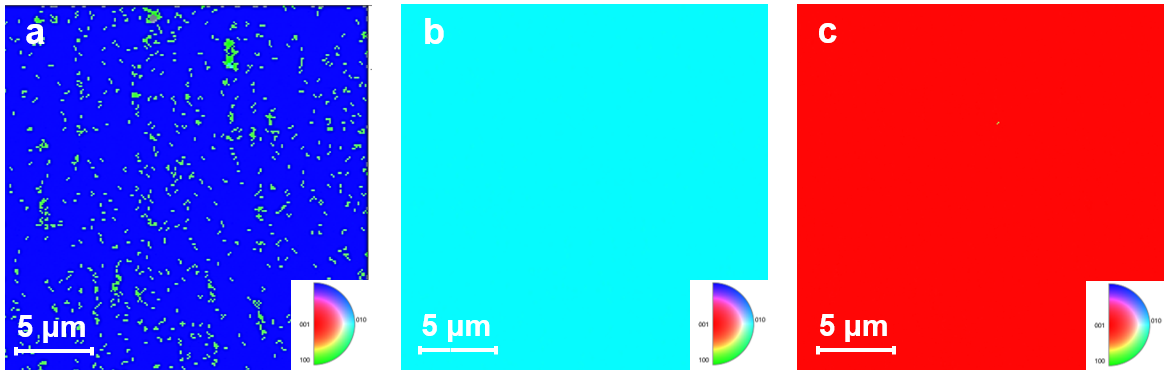
**

**Figure S13:** EBSD maps of epitaxial polycrystalline β-Ga_2_O_3_ at growth temperature below 600°C. a-c) EBSD mapping of the (100), (010), and (001) plane, respectively.

**
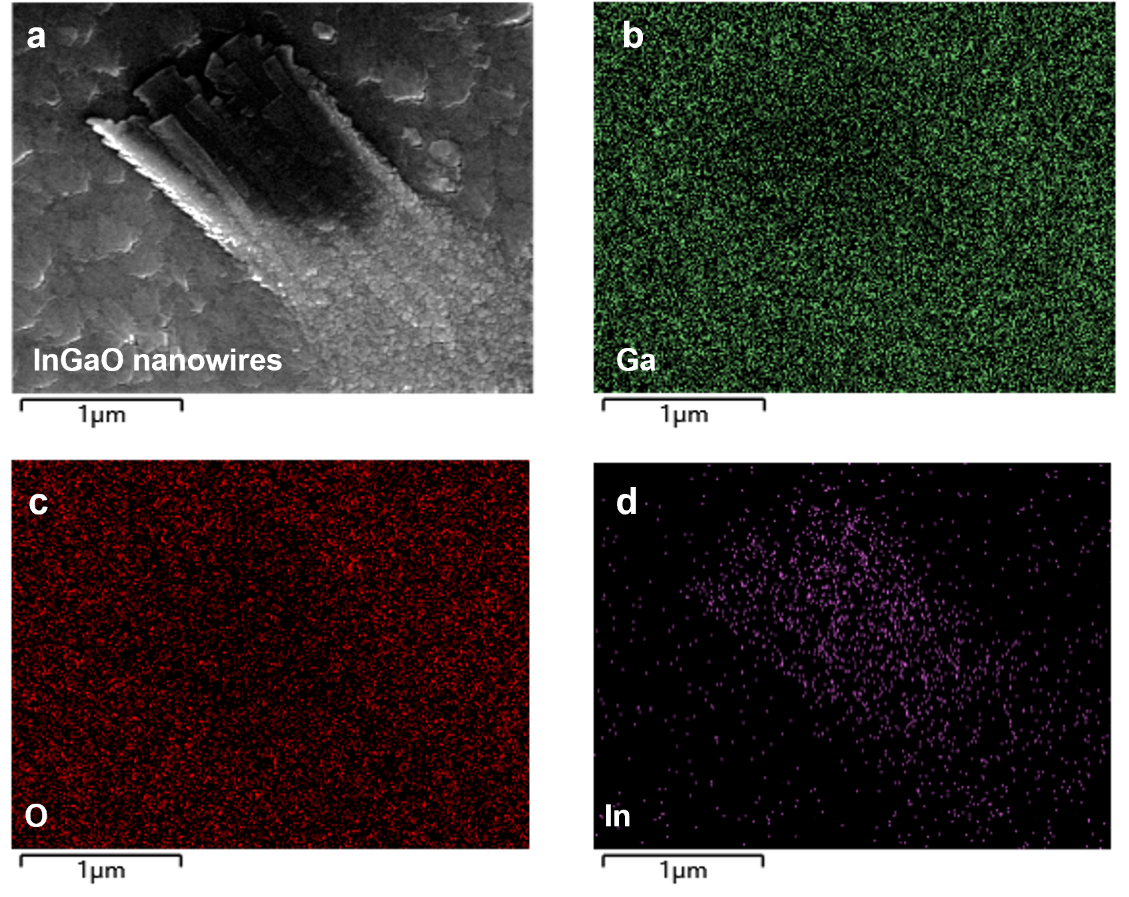
**

**Figure S14:** SEM and EDS images of the InGaO nanowires. a) SEM image of the InGaO nanowires. b-d) EDS elemental distribution mapping for Ga, O, and In, respectively.

**
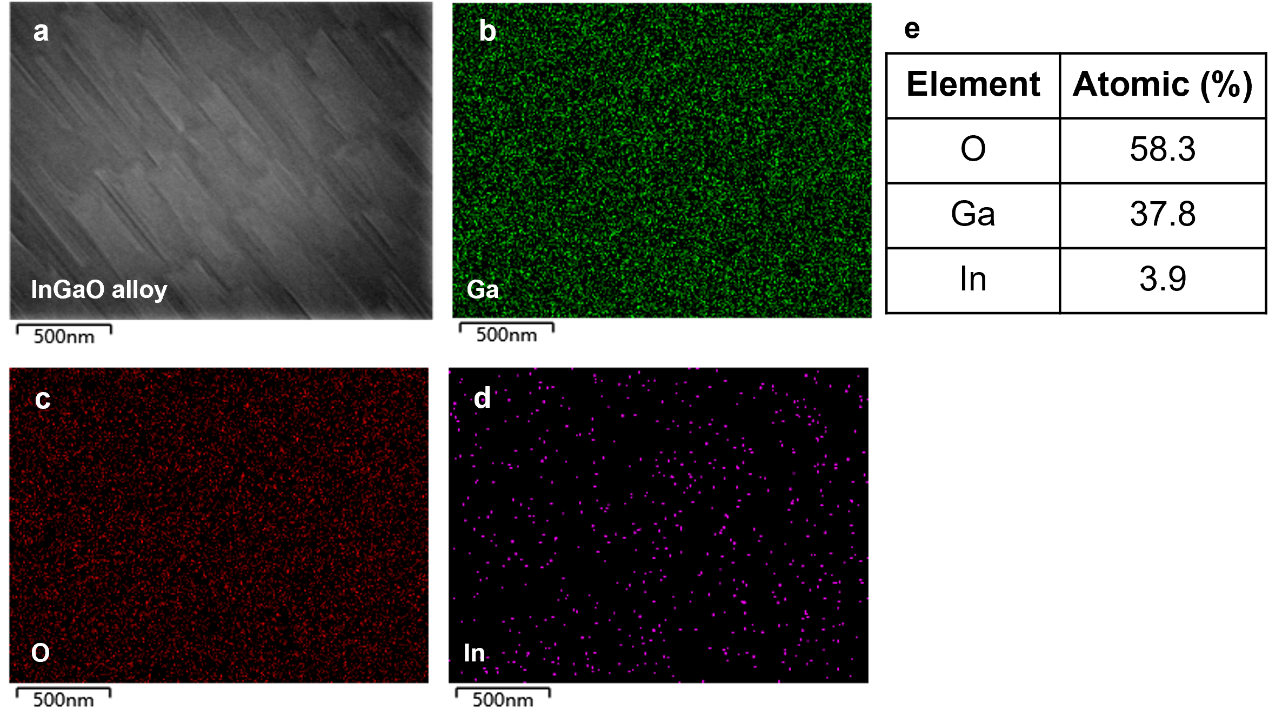
Figure S15:** SEM and EDS images of the InGaO alloyed thin film. a) SEM image of the InGaO alloyed thin film. b-d) EDS elemental distribution mapping for Ga, O, and In, respectively. e) Elemental composition of the InGaO alloyed thin film, showing 3.9% In incorporation in the film.

**
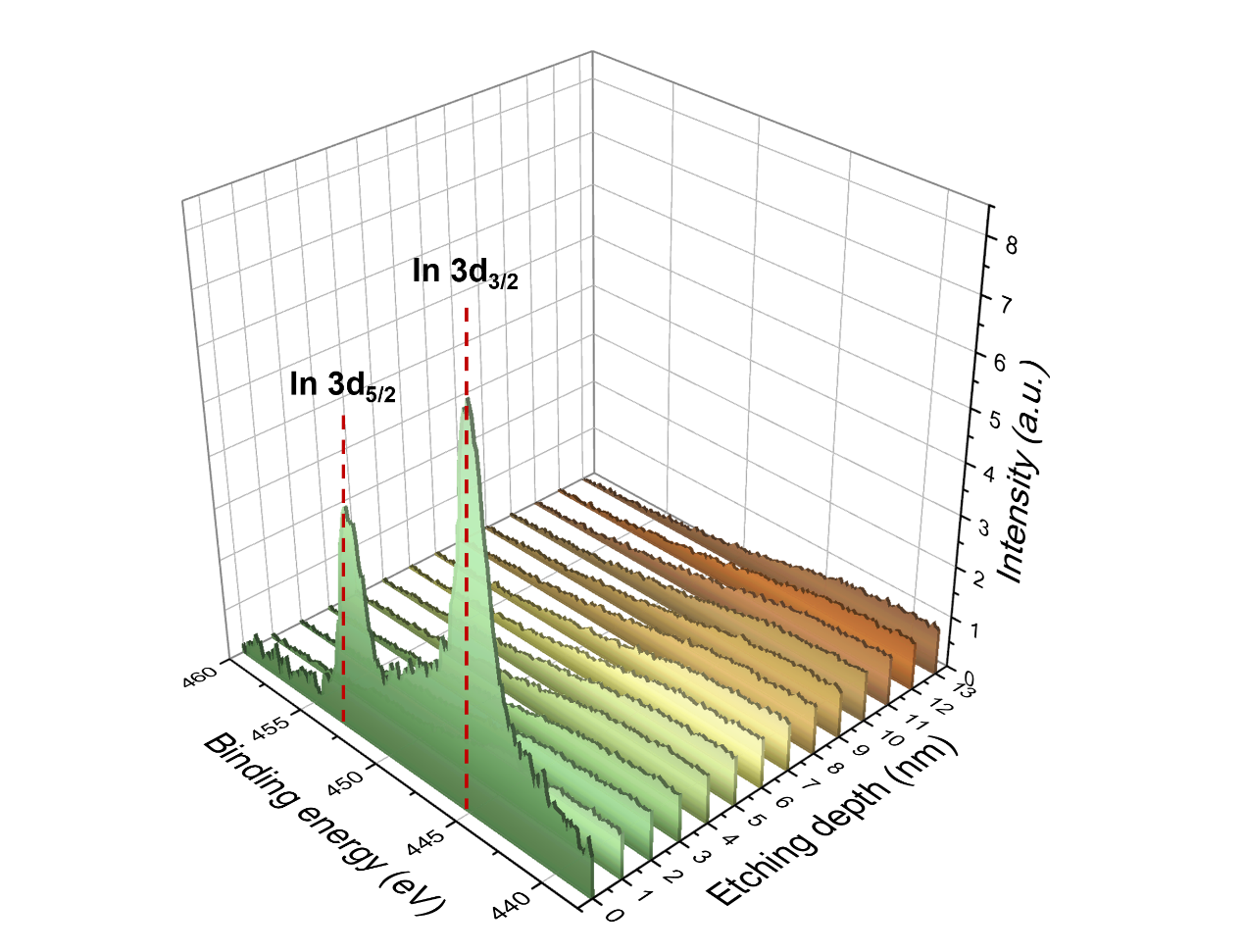
Figure S16:** Depth profiling XPS analysis of single-crystal epitaxial (100) β-Ga_2_O_3_. In was observed to remain on the surface of the film at 0 nm, while no In was detected in the etched film. This observation is consistent with our model, which suggests that In is replaced by Ga, stays on the surface, and desorbs without being incorporated into the film. The surface In can be removed by chemical etching.

**Table S1:** Calculation of Ga atom migration. The activation energy for diffusion, diffusion constants, and diffusion length of Ga atoms on the β-Ga_2_O_3_ (100) surface calculated for different In/Ga ratios using mean-field nucleation theory.

| **In/Ga ratio**  $\boldsymbol{(}\boldsymbol{nm}^{\boldsymbol{-2}}\boldsymbol{s}^{\boldsymbol{-1}}\boldsymbol{/}\boldsymbol{nm}^{\boldsymbol{-2}}\boldsymbol{s}^{\boldsymbol{-1}}\boldsymbol{)}$ | **Nucleation density** $\boldsymbol{(N)}$**(**$\boldsymbol{\mu m}^{\boldsymbol{-2}}\boldsymbol{)}$ | **Activation energy for diffusion**$\boldsymbol{(}\boldsymbol{E}_{\boldsymbol{d}}\boldsymbol{)(eV)}$ | **Diffusion**  **constants**  $\boldsymbol{(D)(}\boldsymbol{cm}^{\boldsymbol{-2}}\boldsymbol{s}^{\boldsymbol{-1}}\boldsymbol{)}$ | **Diffusion length**  $\boldsymbol{(}\boldsymbol{L}_{\boldsymbol{D}}\boldsymbol{)(\mu m)}$ |
| --- | --- | --- | --- | --- |
| 0 | 97.2 | 1.329 | 2.45E-10 | 0.10 |
| 0.06 | 84.25 | 1.230 | 3.76E-10 | 0.11 |
| 0.19 | 12.25 | 0.695 | 1.22E-07 | 0.29 |
| 0.54 | 5 | 0.446 | 1.80E-06 | 0.45 |

**Table S2:** Comparison of diffusion constants of In and Ga atoms on different material surfaces.

| **Materials** | **Substrates and surfaces** | **Temperature**  **(K)** | **Diffusion constants**  $\boldsymbol{(D)(}\boldsymbol{cm}^{\boldsymbol{-2}}\boldsymbol{s}^{\boldsymbol{-1}}\boldsymbol{)}$ | | **Ref.** |
| --- | --- | --- | --- | --- | --- |
| In | Bulk-MoS_2_ | RT | 5.1E-04 | [9] | |
| Ga | GaAs (001) | 1123 | 1.8E-07 | [11,12] | |
| Ga | GaN (001) | 1123 | 5E-14 | [13] | |
| Ga | Ga_2_O_3_ (100) | 1123 | 7E-09 | [11] | |
| Ga | Ga_2_O_3_ (100) | 923 | 2.45E-10 | This work | |
| Ga | Ga_2_O_3_ (100) In-assisted | 1073 | 1.8E-06 | This work | |

**References**

[1] H. Okumura, M. Kita, K. Sasaki, A. Kuramata, M. Higashiwaki,J. S. Speck, *Appl. Phys. Express*. **2014**, *7*, 095501.

[2] J. M. LeBeau, R. Engel-Herbert, B. Jalan, J. Cagnon, P. Moetakef, S. Stemmer,G. B. Stephenson, *Appl. Phys. Lett.* **2009**, *95*, 142905.

[3] M.-Y. Tsai, O. Bierwagen, M. E. White,J. S. Speck, *Journal of Vacuum Science & Technology A*. **2010**, *28*, 354-359.

[4] H. Brune, G. S. Bales, J. Jacobsen, C. Boragno,K. Kern, *Phys. Rev. B*. **1999**, *60*, 5991-6006.

[5] J. A. Venables, G. D. T. Spiller,M. Hanbucken, *Reports on Progress in Physics*. **1984**, *47*, 399.

[6] L. Liu, Z. Chen, L. Wang, E. Polyakova, T. Taniguchi, K. Watanabe, J. Hone, G. W. Flynn,L. E. Brus, *The Journal of Physical Chemistry B*. **2013**, *117*, 4305-4312.

[7] H. Zhou, C. Qiu, Z. Liu, H. Yang, L. Hu, J. Liu, H. Yang, C. Gu,L. Sun, *Journal of the American Chemical Society*. **2010**, *132*, 944-946.

[8] J. D. Thomsen, K. Reidy, T. Pham, J. Klein, A. Osherov, R. Dana,F. M. Ross, *ACS Nano*. **2022**, *16*, 10364-10371.

[9] M. A. Ghani, S. Sarkar, J.-I. Lee, Y. Zhu, H. Yan, Y. Wang,M. Chhowalla, *ACS Applied Materials & Interfaces*. **2024**, *16*, 7399-7405.

[10] M. Wang, S. Mu, J. S. Speck,C. G. Van de Walle, *Adv. Mater. Interfaces*. **2023**, *n/a*, 2300318.

[11] R. Schewski, M. Baldini, K. Irmscher, A. Fiedler, T. Markurt, B. Neuschulz, T. Remmele, T. Schulz, G. Wagner, Z. Galazka,M. Albrecht, *J. Appl. Phys.* **2016**, *120*, 225308.

[12] T. Nishinaga,K.-I. Cho, *Japanese Journal of Applied Physics*. **1988**, *27*, L12.

[13] O. Brandt, H. Yang,K. H. Ploog, *Phys. Rev. B*. **1996**, *54*, 4432-4435.
